# Dry Post Wintertime Mass Surveillance Unearths a Huge Burden of *P. vivax,* and Mixed Infection with *P. vivax* *P. falciparum*, a Threat to Malaria Elimination, in Dhalai, Tripura, India

**DOI:** 10.3390/pathogens10101259

**Published:** 2021-09-29

**Authors:** Ipsita Pal Bhowmick, Tulika Nirmolia, Apoorva Pandey, Sarala K. Subbarao, Aatreyee Nath, Susmita Senapati, Debabrata Tripathy, Rocky Pebam, Suman Nag, Rajashree Roy, Dipanjan Dasgupta, Jayanta Debnath, Kongkona Gogoi, Karuna Gogoi, Lakhyajit Borah, Rajdeep Chanda, Arup Borgohain, Chelapro Mog, Ujjwal Sarkar, Phiroz Gogoi, Bishal Debnath, Jyotish Debbarma, Dibya Ranjan Bhattacharya, Pyare Lal Joshi, Harpreet Kaur, Kanwar Narain

**Affiliations:** 1 Regional Medical Research Center-Northeast Region (RMRC-NE)-ICMR, Dibrugarh 786001, India; tulikanirmolia@gmail.com (T.N.); senapatitiku999@gmail.com (S.S.); debabrata.tripathy07@gmail.com (D.T.); snag8540@gmail.com (S.N.); royrajashree6@gmail.com (R.R.); dipanjandasgupta89@gmail.com (D.D.); debnathjayantabsc@gmail.com (J.D.); koko1.gogoi@gmail.com (K.G.); karunarmrc@gmail.com (K.G.); lakhyaborah01@gmail.com (L.B.); chelapromog348@gmail.com (C.M.); ujjwalsarkar188@gmail.com (U.S.); phirozgogoi@gmail.com (P.G.); bishaldebnath89@gmail.com (B.D.); jd45683968@gmail.com (J.D.); drbhattacharyya@yahoo.com (D.R.B.); kanwar_narain@hotmail.com (K.N.); 2 Indian Council of Medical Research (ICMR), Ramalingaswami Bhavan, Delhi 110029, India; apoorva.icmr@gmail.com (A.P.); kaur.hq@icmr.gov.in (H.K.); 3 Formerly National Institute of Malaria Research-ICMR, Delhi 110077, India; subbaraosk@gmail.com; 4 Northeastern Space Applications Centre, Department of Space, Government of India, Umiam 793103, India; aatreyeen04@gmail.com (A.N.); rocky.pebam@gmail.com (R.P.); arupborgohain@gmail.com (A.B.); 5 Mizoram University, Aizawl 796004, India; rajdeepchanda1991@gmail.com; 6 Formerly National Vector Borne Disease Control Program (NVBDCP), Delhi 110054, India; doctorjoshi00@gmail.com

**Keywords:** malaria, *Plasmodium vivax*, *Plasmodium falciparum*, mixed-infections, mass surveys, rapid diagnostic kits, molecular diagnostic technique, dry winter season, asymptomatic malaria

## Abstract

With India aiming to achieve malaria elimination by 2030, several strategies have been put in place. With that aim, mass surveillance is now being conducted in some malaria-endemic pockets. As dry season mass surveillance has been shown to have its importance in targeting the reservoir, a study was undertaken to assess the parasite load by a sensitive molecular method during one of the mass surveys conducted in the dry winter period. It was executed in two malaria-endemic villages of Dhalai District, Tripura, in northeast India, also reported as *P. falciparum* predominated area. The present study found an enormous burden of Rapid Diagnostic Test negative malaria cases with *P. vivax* along with *P. vivax* and *P. falciparum* mixed infections during the mass surveillance from febrile and afebrile cases in dry winter months (February 2021–March 2021). Of the total 150 samples tested, 72 (48%) were positive and 78 (52%) negative for malaria by PCR. Out of the 72 positives, 6 (8.33%) were *P. falciparum*, 40 (55.55%) *P. vivax*, and 26 (36.11%) mixed infections. Out of 78 malaria negative samples, 6 (7.7%) were with symptoms, while among the total malaria positive, 72 cases 7 (9.8%) were with symptoms, and 65 (90.2%) were asymptomatic. Out of 114 samples tested by both microscopy and PCR, 42 samples turned out to be submicroscopic with 4 *P. falciparum*, 23 *P. vivax*, and 15 mixed infections. Although all *P. vivax* submicroscopic infections were asymptomatic, three *P. falciparum* cases were found to be febrile. Evidence of malaria transmission was also found in the vectors in the winter month. The study ascertained the use of molecular diagnostic techniques in detecting the actual burden of malaria, especially of *P. vivax*, in mass surveys. As Jhum cultivators in Tripura are at high risk, screening for the malarial reservoirs in pre-Jhum months can help with malaria control and elimination.

## 1. Introduction

According to the World Malaria Report 2020, India contributed to the largest reductions in malaria cases in South East (SE) Asia from ~20 million cases in 2000 to ~5.6 million in 2019. However, it still accounted for 86% of all malaria deaths of the SE Asia region [1].

India is aiming for malaria elimination by 2030, and a National Strategic Plan (NSP) has been taken up accordingly. According to the National Strategic Plan (NSP 2017-22), districts of India have been stratified into four distinct categories in terms of transmission intensity, which is measured by the annual parasite incidence (API). Category III Districts, the high transmission areas with API > 2, are in an intensified control stage and need concerted efforts to bring down the disease burden [2]. Many of the districts of NE states in India, such as Tripura, Meghalaya, and Mizoram, are in Category III. A few pockets inside such districts could be regarded as extreme areas, having disproportionately high API values (>10), with hilly forested tribal dominated regions largely contributing to malaria. Mass screening is considered as one of the strategies by the National Vector Borne Disease Control Program (NVBDCP) surveillance to reduce the vast transmission reservoir. Accordingly, several cross-sectional mass surveys were conducted in malaria-endemic regions of NE India in the last two years. The hilly, forested, and tribal-dominated areas of NE India continue to be malaria-infested and are prone to outbreaks. Tripura is one such state where a major outbreak occurred in 2014, affecting >50,000 people and causing ~70 deaths, most of whom were children [3] and again one in 2018. Several endemic villages of Tripura were selected by national and state malaria programs for the cross-sectional surveys during 2019–2020. This year (2021), several such surveys were planned; these included one in the dry post-winter season, February to March 2021.

Studies have shown that in the dry season, when the clinical incidence of malaria is generally low, malaria cases are found with low parasitemia and are asymptomatic. As clinical malaria is seasonal and mainly in the rainy season, subclinical *P. falciparum* infections persist throughout the dry season, playing the role of the reservoir infections for the transmission season, which would be several months later [4,5,6].

Host survival during the dry months is considered essential to resume *P. falciparum* transmission in the subsequent rainy season. The parasite has been thought to have evolved strategies for preventing potentially fatal host pathology. It ensures persistence during periods when there are few or no mosquito vectors [7]. In Sudan, it has been demonstrated that low-density asymptomatic infections perpetuate transmission over the dry season [4].

Additionally, parasite reservoirs are thought to be associated with low-density asymptomatic infections. There is a clear evolutionary advantage to the parasite for sustaining it, since asymptomatic individuals do not seek treatment and represent a parasite reservoir from which malaria vectors can become infected [8,9]. In some studies, these reservoirs of asymptomatic cases, as found by the maps of PCR-detected *P. vivax* infections by households, are fewer and more tightly clustered during the dry season, while they expand during the wet season with the expansion of the breeding ground [10]. Another study found clusters of *P. vivax* infections in the dry season, where only one symptomatic case of malaria was found by Rapid Diagnostic Test (RDT), indicating that the observed clusters are almost exclusively composed of asymptomatic carriers. It was also shown that groups of homesteads consisting of asymptomatic carriers could act as stable clusters for several years. These asymptomatic, potentially long-term infections might be sufficient for sustaining malaria transmission and therefore can be targeted for malaria control and elimination [11]. Hence, finding the asymptomatic, submicroscopic cases in the dry season can be potential targets for cutting the transmission chain. A study in Bangladesh showed that malaria prevalence in the winter survey (dry season) was not significantly lower than that in the summer survey where both *P. falciparum* and *P. vivax* were found, and most cases were asymptomatic [12].

With this background, this study was planned to assess the burden of malaria infections and whether asymptomatic and submicroscopic malaria cases are present in dry post-winter months in malaria-endemic pockets of Dhalai district in Tripura. Dhansinghpara and Bidyapara villages, under the Gurudhanpara sub-center (SC), were amongst the highly malaria-endemic pockets in Dhalai and chosen by the National and State Malaria Programs for mass survey in February 2021–March 2021. The detection method used in the survey was RDT. Considering that RDT might miss asymptomatic submicroscopic cases, mostly with low parasitemia, this study was planned to use more sensitive nested PCR. A subset of samples was also screened by microscopy.

## 2. Results

### 2.1. Prevalence of Malaria by 18S Nested PCR

A total of 150 samples, 90 from Dhansinghpara and 60 from Bidyapara, were tested by 18S nested PCR, and 72 people out of them were found positive with a total malaria case positivity of 48% (95% CI, 40.16–55.94), with 52.2% (95% CI, 42.02–62.24) in Dhansinghpara and 41.7% (95% CI, 30.02–54.27) in Bidyapara. Details of these infections and species distribution are given in Table 1.

### 2.2. Symptomatic and Asymptomatic Cases

Among the total of 150 samples tested, 13 (8.7%) were with symptoms, and 137 (91.34%) were without any symptoms. Out of those 150 tested, 72 were malaria positive, and among the positives, 65 (90.3%) were asymptomatic cases and seven (9.7%) were symptomatic cases. Out of 78 negative samples, 6 (7.7%) were with symptoms, while among the total malaria positive cases, 90.3% were asymptomatic.

There were seven symptomatic cases, of which four were *P. vivax,* and three were *P. falciparum* + *P. vivax*. There were 65 asymptomatic cases, of which 6 were *P. falciparum*, 36 *P. vivax*, and 23 *P. falciparum* + *P. vivax*. Species wise age group distribution details with % for each age group are given in Table 2.

The only case found positive by RDT was *P. falciparum*, while it was *P. vivax* by both microscopy and 18S PCR.

Out of 150 total samples, 65 (43.3%) were asymptomatic positive and 7 (4.7%) symptomatic positive cases, as found by PCR. Out of 78 negative samples, 6 (7.7%) were with symptoms (Table 2), while among the total malaria positive cases, 90.3% were asymptomatic.

Chi square analysis was conducted to test any association between symptomatic and asymptomatic cases with age. However, no such associations were found. Interestingly, asymptomatic cases occurred in younger age groups as well. However, there was a distinctly decreasing proportion of symptomatic cases from 9.09 to 6.25 to 2.67% as age groups increased from 0 to <5, 5 to <15, and 15 and above years (Table 2).

### 2.3. Submicroscopic Cases and Parasite Density in Microscopically Identified Cases

Out of a total of 150 samples, 114 samples, namely 67 from Dhansinghpara and 47 from Bidyapara, were tested by both microscopy and 18S PCR. Among them, 11 were positive by both methods: two *P. falciparum*, two *P. vivax*, and seven mixed. Out of 114 samples tested, 42 samples were found to be submicroscopic as they were negative by microscopy, while PCR found them positive, which were 4 *P. falciparum*, 23 *P. vivax*, and 15 *P. falciparum* + *P. vivax*. In Dhansinghpara these were 3 *P. falciparum*, 16 *P. vivax*, and 3 *P. falciparum* + *P. vivax*, and in Bidyapara, 1 *P. falciparum*, 7 *P. vivax* and 9 *P. falciparum* + *P. vivax* cases.

Two samples were detected as *P. falciparum* + *P. vivax* in PCR but only *P. vivax* by slide; hence, they were considered as submicroscopic *P. falciparum* cases. One sample was detected as *P. falciparum* + *P. vivax* in PCR but only *P. falciparum* by slide; hence, it was considered as a submicroscopic *P. vivax* case. Age-group distribution of species-specific submicroscopic case details is given in Table 3. No significant association with any age group was found for the submicroscopic cases. It is important to note the presence of submicroscopic *P. vivax* and mixed infections in the less than 5 years age group.

Among the submicroscopic cases, 39 were asymptomatic (4 *P. falciparum*, 22 *P. vivax*, 13 *P. falciparum* + *P. vivax*), and 3 were symptomatic (1 *P. vivax*, 2 *P. falciparum* + *P. vivax*).

While all three submicroscopic symptomatic infections were in the 5–15 year age group, there were four submicroscopic asymptomatic cases in the <5 year age group.

It is important to mention here that none of the people tested received an anti-malarial two weeks before the blood was taken for PCR. Hence, the submicroscopic infections obtained could not be due to drug treatment.

Asexual densities calculated for some cases where microscopy could detect the parasites were in the low to moderate range for both *P. falciparum* (80–600 parasites/µL) and *P. vivax* (90–720 parasites/µL).

### 2.4. Risk Factor Analysis for Malaria Cases

No significant risk (*p* > 0.05) for symptomatic and asymptomatic cases was found to be associated with age groups, occupation, or long-lasting insecticidal net (LLIN) use by logistic regression analysis.

### 2.5. Ecological Characteristics of the Area and Distribution of the Cases

**Figure 1:**
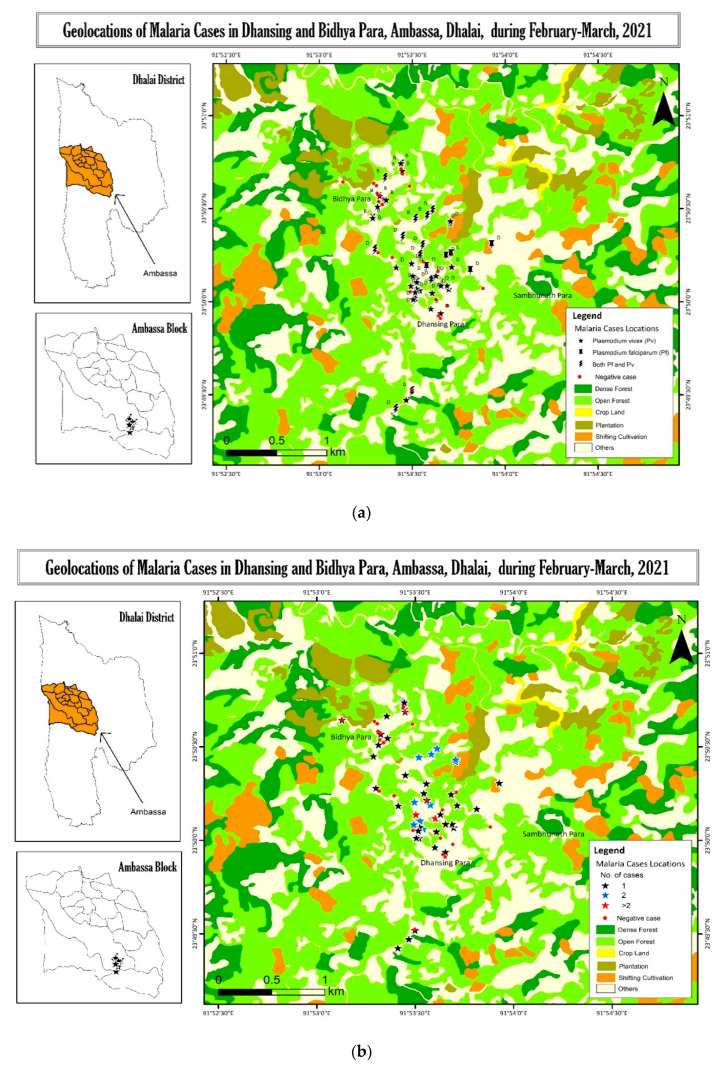

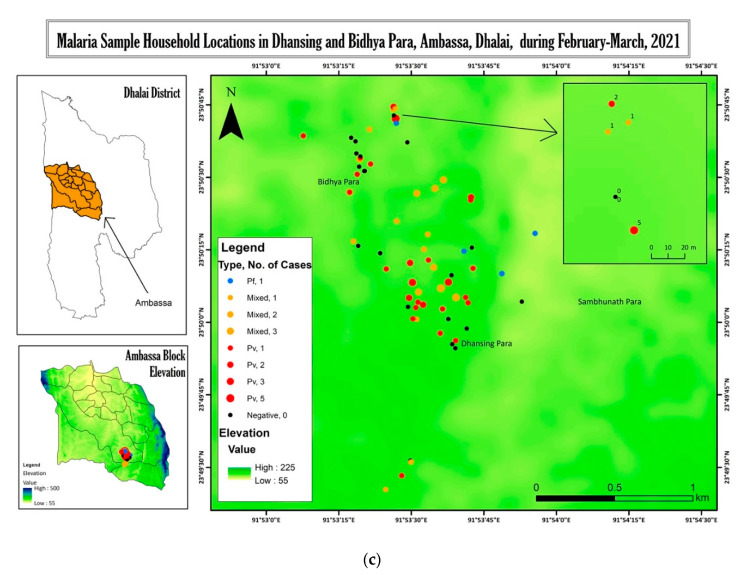
(**a**) Ecological map showing the geolocation of different species of PCR positive malaria cases in Dhansinghpara and Bidyapara along with the negative cases. (**b**) Ecological map showing malaria cases, plotted by the number of cases per household in Dhansinghpara and Bidyapara along with the negative cases. (**c**) Graphical representation of the locations of positive and negative case households, which show that there are several households with a high number of cases. The size of the circle indicates the number of cases per household (0–5). Elevation is also represented. Some cases have overlapped in the map; one such area is magnified in inset.

The ecological maps (Figure 1) showed that the study area terrain is low-lying hills (80–400 m above mean sea level) dominated by forests and croplands in the flat valley in-between the hills. They are surrounded by open forests and shifting cultivation (Jhum) fields close to the dense forests. Dense forests are dominated by trees with a canopy density of >40%. Open forests have canopy densities between 10% and 40%. These are the land-uses that have regenerated from the open or dense scrubland and shifting cultivation, i.e., burn and slash (Jhum) fallow area, etc., during the past few years and may become a dense forest in the due course of time. Shifting cultivation (Jhum) fields are located around the habitation area within a distance of 1–1.5 km. The plantations are mostly planted in uniform close spacing, mostly with teak (*Tectona grandis*) and rubber (*Hevea brasiliensis*); hence, they have a closed canopy. There was less undergrowth, and both the trees are deciduous in nature. Croplands are confined only to the narrow valley area among the hilly terrain and were predominant with paddy cultivation. Cases in households showed that there were several households with more than one case, and those households were found to be located in proximity to each other and were in the vicinity of the forest areas.

### 2.6. Entomological and Meteorological Findings: Month-Wise Anopheles Density Plotted with Monthly Total Rainfall; Monthly Mean, Minimum, and Maximum Temperature; Mean Relative Humidity; and NDVI

**Figure 2:**
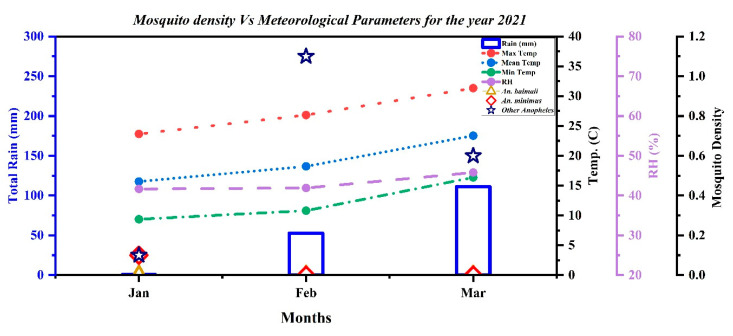
Month-wise vector (*Anopheles minimus* and *Anopheles baimaii*) abundance from CDC light trap collections plotted with monthly total rainfall; monthly minimum, maximum, and mean temperatures; and mean relative humidity for January to March 2021 for the.

As shown in Figure 2, there was almost no rainfall in January and little rainfall in February and March 2021, with the relative humidity being less than 50% in all three months. We also plotted the API found during the study months and for one month preceding the start of the mass survey. *Anopheles* species abundance is the number of specimens of a species obtained per the Centre for Disease Control and Prevention (CDC) miniature light trap per night.

Only a single specimen of *Anopheles minimus* was found in these two villages during this time period (found in January). Other *Anopheles* found were three specimens of *A. jeyporensis*, six *A. barbirostris,* four *A. Varuna* and four *A. Maculatus*, and one *A. annularis*.

*A. minimus* tested for *Plasmodium* sporozoite positivity by the molecular method in the dissected head and thorax part was found positive for *P. vivax*.

## 3. Discussion

The present study found a staggeringly large number of malaria cases and a high proportion of *P. vivax* along with *P. vivax* and *P. falciparum* mixed cases among them. These were not detected by RDT during the mass surveillance from febrile and afebrile people in dry winter and spring months in two malaria-endemic villages of Dhalai District. While many cases are reported yearly in the study area, cases are very high during monsoon and post-monsoon times. A few cases are found in routine active and passive surveillance from January to March. Analysis of data of Ambassa PHC under which the study villages fall shows 2–5% of yearly cases in January to March, as evident from the data of 2018–2021 (Supplementary Table S1a). Yearly case data for 2019 and 2020 include mass surveillance data from the program conducted in monsoon and post-monsoon seasons. Similar analysis of yearly and monthly malaria case data for the study villages Bidyapara and Dhansinghpara showed 5–10% of annual cases during January to March (Supplementary Table S1b). Very few (six) malaria cases were reported during February and March 2021 from these two villages, even when the malaria program undertook mass screening using RDT. Ambassa PHC cases, as reported by the program, also showed a similar ratio of cases during January–March.

In contrast, this study showed a considerable number of cases with a very high prevalence of ~48%, especially of *P. vivax* and mixed infections during this dry period, which totally remains undetected by routine active and passive surveillance and even by mass surveillance if only RDT is used. There are even cases beyond microscopic detection. While these submicroscopic cases are most likely low-density ones (<50 parasites/µL), and the majority of the cases detected by microscopy were found to have <100 parasites/µL, there were a few microscopically detected cases with a higher density, such as 600–720 parasites/µL, yet they were undetected by RDT for both parasite species. In the case of *P. falciparum*, these cases might have HRP II/III gene deletion, which is worthy of future exploration. In the case of *P. vivax*, this might indicate a lower sensitivity of RDT kits in field samples of this area, which again needs to be investigated in future. Furthermore, while the majority of these cases were asymptomatic, some of them were symptomatic as well, which were submicroscopic.

Interestingly, most *P. vivax* and mixed infections found in this study were asymptomatic in all the age groups, i.e., in below 15 years and more than 15 years groups. While there was no significant association of the symptomatic infections with the age group, the proportion was higher in the <5 age group (Table 2)

Unusually high *P. vivax* cases were detected from a subset of the samples analyzed for malaria. Although the sample size was not very big, we covered a considerable portion of the population (we covered 373 people by RDT, which is >55% of the population of 679 of these two villages who were considered for mass survey) in the survey in an unbiased manner. The survey was attempted to include all the people who were available and willing to give blood, irrespective of their febrile status.

This study thus warrants the use of molecular techniques in the mass survey for detecting parasite positives, especially *P. vivax,* and the importance of screening in the dry season, which can assist in determining the course for control and elimination of malaria. Previously, a study conducted during winter in Tripura after the epidemic had shown a high prevalence of low-density asymptomatic cases (unpublished data).

Although the infectivity of the asymptomatic and submicroscopic cases was not tested in this study, several reports [8,9,10] have shown the infectivity of asymptomatic low-density infection of both the parasite species. Especially the study in Thailand, which showed the presence of a low-density infectious reservoir of *P. vivax* as clusters, raises the possibility of these infections serving as the infectious reservoir. More direct proof of transmission in cold, dry months comes from our finding on the presence of parasites in *A. minimus* in January. Although traditionally it has been thought that temperatures below 18 °C would not be conducive to parasite development in the vectors, recent studies are challenging this notion. Waite et al. (2019) [13] showed the feasibility of schizogony, though a prolonged one up to 16 °C. As their research involves laboratory-adapted mosquito and parasite strains, they have stressed the need for a more detailed assessment of other parasite strains and species of malaria parasites from the field and exploring studies with local mosquito–parasite pairings. These studies would be worthwhile to assess the extent of malaria transmission in low temperatures. There have been reports of mosquitoes having transmission in an amicable environment like indoor or other places, which would not be that cold in the cold months. It is very much possible that the vectors find local microenvironments where the temperature is not low in the cold months, and thus, they sustain the infection in the dry cold seasons.

There have been reports of some mass surveillance studies from other parts of India. One study [14] in Chennai (Tamil Nadu), Nadiad (Gujarat), and Rourkela (Odisha), during 2012–2015, where the study period included dry winter and post-winter seasons as well, showed asymptomatic infections ranging from 21% in Gujarat and 64% in Rourkela to 71% in Chennai, and different proportions of parasite species found in different study sites among asymptomatic, submicroscopic carriers, and age groups but very few mixed infections. A survey conducted in Chhattisgarh in 2016 during the low transmission season detected ~35% submicroscopic and ~77% afebrile malaria cases. The sensitivities of RDT, microscopy, and PCR were 53.3, 47.5, and 87.4%, respectively, showing that a significant number of infections can be missed during routine testing by RDT and microscopy [15]. Another study in Balaghat, Madhya Pradesh, showed the relevance of afebrile parasitemia due to both *P. falciparum* and *P. vivax* [16]. A survey in June 2012 from Purulia district of West Bengal on asymptomatic and submicroscopic infections detected only ~1% submicroscopic infections in 963 participants while detecting five infections of *P. falciparum* and none of *P. vivax,* which were not seen by RDT [17]. A cross-sectional survey conducted during May–June 2017 in the Kandhamal district of Odisha reported [18] that of ~30% randomly selected samples analyzed using real-time PCR, the following species-specific prevalence by qPCR among asymptomatic positive cases was found: 57% *P. falciparum*, 29% *P. vivax*, and 14% *P. falciparum* + *P. vivax*.

However, there have been few such studies in NE India, which has multiple malaria-endemic pockets. A previous study in 2014 in dry winter months (January–February) in the Missamari area of Assam, situated in northeast India, found no positive cases by RDT, microscopy, or PCR [19]. Although a similar PCR method was used in that study, it may be mentioned that dry blood spots were used in that study while whole blood was used in this study.

This study found a considerably higher positivity rate in RDT negative samples than all other studies reported in India. Among them, the proportion of *P. vivax* and mixed infections was also much higher, and that too in the dry post-winter month, which is not typical malaria season.

The NE states were shown to be co-endemic for *P. falciparum* and *P. vivax* species. High proportions of *P. vivax* cases (60–80%) were seen in Arunachal Pradesh and Nagaland in the north with the alpine environment, 42–67% in Manipur, whereas in Assam, they varied from 23 to 31% with a subtropical and tropical climate. On the other hand, Tripura, with a warm and humid tropical climate and large stretches of tropical evergreen, semi-evergreen and moist deciduous forest and Meghalaya and Mizoram, had the lowest proportion of *P. vivax* cases [20].

In Tripura, *P. falciparum* has been consistently the dominating species as per the clinical cases reported by the national malaria program [21]. Traditionally, *P. vivax* malaria cases have been reported as very low by NVBDCP for the whole of Tripura, where the results are primarily RDT-based and only to some extent on microscopic examination while being performed exclusively on the symptomatic febrile cases. The same can be seen from the data from 2008 to 2019 (Supplementary Table S2), where less than 10% of the total malaria cases (ranging from 3.1 to 9.5%) of *P. vivax* were detected. However, for the first time, this study reports the presence of a substantial asymptomatic and submicroscopic burden of *P. vivax* infections singly or mixed with *P. falciparum* in NE India. This study shows the importance of conducting similar cross-sectional surveillance studies using molecular methods in other areas of NE India to determine the real burden of *P. falciparum* and *P. vivax* cases.

Once regarded as a relatively benign disease, *P. vivax* malaria is now acknowledged as a significant public health concern leading to life-threatening complications, miscarriage, chronic infection, and increased mortality. A growing list of evidence challenges the previously-existing notion of *P. vivax* being benign [22,23,24]. Additionally, *P. vivax* poses specific difficulties to elimination, mainly due to its ability to relapse weeks to months after the initial infection. It is responsible for a significant burden of malaria worldwide, accounting for half of all the cases in Asia and Latin America [25]. According to the 2019 World Malaria Report, of the total global *P. vivax* burden, 53% is in the WHO South-East Asia Region, and India alone accounts for 47% of this burden [26].

*P. vivax* is known to cause lower parasite densities than *P. falciparum*. In many cases beyond the limit of RDT detection or even microscopic detection leading to high rates of false-negative *P. vivax* symptomatic cases [27,28]. In Columbia, ~56% of the asymptomatic *P. vivax* carriers were infective to *A. albimanus* mosquitoes [29]. Another study involving asymptomatic volunteers from Thailand using *P. vivax* and *A. dirus* mosquitoes showed 13% samples were infective [8]. In both these studies, low-density asymptomatic *P. vivax* was infective, thus establishing their role in the transmission.

There is a possibility that some of the *P. vivax* cases detected in this study are relapse cases, as the transmission may not be high during this period, which is evident from the *Anopheles* density found in the entomological surveillance. This can be indicative of the absence or incomplete treatment of primaquine. As all the *P. vivax* infections, symptomatic or asymptomatic if left untreated, go undetected in routine surveillance programs where RDT/microscopy is used, it is quite probable that a large proportion of *P. vivax* remains as hypnozoites in the liver, which can later relapse. These hypnozoites can become spontaneously activated and induce asymptomatic periodical infection or clinical episodes [30]. It has been shown that dormant *P. vivax* hypnozoites can activate at variable periodicity depending on geographical region [31], which means that untreated sub-microscopic infections can continue to relapse in the future. In addition, these parasites can cause multiple clinical attacks over the months (up to approximately two years) following a single infectious bite by the *Anopheles* vector species. This can potentially carry on the transmission with each relapse.

Hence, the determination of accurate *P. vivax* burden, including the low-density asymptomatic burden, is important from the malaria control and elimination point of view in the known *P. falciparum* endemic areas as well. However, most research and published literature on malaria still focuses on *P. falciparum* and much less on *P. vivax* [22]. So far, the data on the *P. vivax* sub-microscopic burden is limited in India, with almost no available data from NE India.

*P. falciparum* malaria has receded in many regions in Asia and South America. In contrast, *P. vivax* malaria has remained a harder challenge as it responds much more slowly to the established control methods that have driven *P. falciparum* to near elimination in those areas [32]. In India, a similar trend can be found upon analysis of the incidence of *P. falciparum* and *P. vivax* for the past few years (Supplementary Figure S1). It shows that although the overall malaria clinical burden has been dropping over the last decade, it is mainly the burden of *P. falciparum* that is decreasing while *P. vivax* is not. This can very well be the situation of states like Tripura. This might be attributed to the non-detection of low to moderate density *P. vivax* malaria in traditionally known *P. falciparum* areas and incomplete treatment of *P. vivax* cases, leading to relapses and a considerable burden of low-density asymptomatic infections.

Malaria elimination efforts are being ramped up in India and worldwide to eliminate the disease by 2030. From this perspective, the findings of this study are significant, which showed a very high hidden burden of *P. vivax* and mixed cases, most of which are sub-microscopic and asymptomatic in *P. falciparum* predominant area. This study also suggests that sensitive diagnostic methods like PCR, other than microscopy, may be used for mass surveillance, especially in the dry post-winter season, to unearth the reservoir of infection. Ecological maps (Figure 2) show that the cases are in the vicinity of the forested regions and Jhum cultivation lands, and there are several households with 2–5 cases. Some of these households are located in proximity to each other. As the maps are constructed from the satellite images of 2019, and the shifting cultivation, open forest, and dense forest areas are interchanged among themselves, we have not associated the proximity of cases to any particular forest type. Still, it can be seen that they are in close vicinity to the forest land. It would be worth finding out whether there are clusters and targeting them with intervention [33] and treatment aimed at malaria control and elimination. These reservoirs can fuel transmission in the subsequent months. It is all the more relevant for these areas as most of the people in the study area are Jhum cultivators. Jhum cultivation has been shown as a risk factor for malaria in adjacent Bangladesh [34] and Tripura as well [35]. Jhum cultivation involves shifting cultivation in the deep forested areas, which involves the temporary stay of the cultivators or their majority of time being spent in the forests from April onwards, reducing their availability to health workers. Therefore, screening for the reservoir in pre-Jhum months (February to mid-April) and treating the positive cases with antimalarial drugs may help reduce transmission.

## 4. Methodology

### 4.1. Study Site and Population

Tripura is one of the smallest states among the eight states of northeast India that shares 84% of its total border with Bangladesh. Dhalai is the largest district of the state, situated approximately between longitude 91° 51.608″ E and latitude 23° 55.501″ N with a population of 378,000 according to 2011–2018 census data. Dhansinghpara and Bidyapara villages are located at 23°49′55″ N latitude, and 91°53′33″ E longitude, respectively, and about 155 m above mean sea level (MSL) and about 12 km south of the town of Ambassa, which is the district headquarters. About 40% of the area is under forest cover, while 45% is under scrubland. Agriculture land use covers about 0.13%, while about 6% of the site is under shifting cultivation.

### 4.2. Community Mass Survey

A door-to-door and health camp-based mass survey was carried out in Gurudhan subcenter under the Ambassa Primary Health Centre (PHC) in Dhalai District, from the third week of February 2021 to the first week of April 2021, by state health workers and volunteers. As these two villages are high endemic villages in the area (Supplementary Table S1), these two villages were chosen to study malaria burden, symptomatic and asymptomatic cases, and plasmodium species distribution in dry winter months.

Health camp-based mass surveys are those where health camps are organized by the health department in the village premises, and villagers are informed beforehand to come and get tested, irrespective of fever status. Regarding the choice of household or individuals, the aim of the survey, as decided by Health Department and Malaria Program was to cover as many villagers as possible, if not all. Thus, whoever was available for testing and willing to get tested was tested and treated.

The total population in the two villages was 1009, namely 626 in Dhansingh and 383 in Bidyapara. Anyone available during the survey time and willing to give blood, irrespective of the presence of symptoms, was recruited for taking blood. There were some villagers who were not present in the village for a more extended period, either for a job or study or other reasons. They were not considered for the mass survey. Total such temporary absentees were 330: 203 in Dhansinghpara and 127 in Bidyapara. Hence, a total of 679 people was considered for the mass survey. During our survey in this period, among the available people, a total of 104 refused, namely 71 in Dhansinghpara and 33 in Bidyapara. Thus, 373 people (~55% of the considered population) could be covered, and blood was taken from them to test RDT. The rest of the people who live in the villages but were not found during the survey as they had gone out to the forest for Jhum cultivation or some other work were termed as unavailable. This year, due to elections in this area and other unrest, evening time visits were not possible, which are usually undertaken while conducting a mass survey to reach people who are unavailable during the daytime. However, as it could not be done this year, there was a high number of unavailable people. Questions on age, relations to HH head, fever history, travel history, forest exposure history, symptoms, and vector control measures were asked during the mass survey. In the case of febrile patients, detailed questionnaires were filled up.

### 4.3. Blood Sample Collection

Finger-prick blood samples were collected from individuals irrespective of the febrile status. Finger-prick blood samples were used for microscopic slide preparation and collected in small tubes with EDTA powder for molecular technique. Written informed consent from the participant or the guardians in case of minors was obtained. Blood spots were also taken on filter paper, air-dried, and stored at 4 °C for future molecular analysis.

All the 373 samples collected were tested by RDT by the Malaria Program. Blood slides and blood spots on filter papers were taken, and also whole blood was taken from the same finger prick in a tube containing EDTA, wherever available. For only 150 samples could whole blood be collected in the tubes. From the others, finger-prick blood could not be collected in tubes, as sufficient blood was not there. Therefore, in this study, we conducted PCR on these 150 whole blood samples collected in the tubes, not on the dried blood spots.

### 4.4. Rapid Diagnostic Test (RDT)

An RDT was performed only on febrile cases (people complaining of fever in the last seven days). Blood samples were collected to detect the presence of *P. falciparum* and *P. vivax* parasites using an RDT Malarigen kit (Aspen Laboratories) supplied by NVBDCP, India, according to the manufacturer’s protocol.

### 4.5. Blood Slide Examination (BSE)

Both thick and thin blood smears were examined. Thick smears were hemolyzed, and thin smears were fixed with methanol and stained with 10% Giemsa stain. Stained blood smears were examined under 100X oil immersion using a Zeiss microscope by three experienced technicians to confirm the presence of the *Plasmodium* parasite [36].

### 4.6. Parasite DNA Extraction

Parasite DNA was extracted from the blood samples that were collected in 0.5 mL microcentrifuge tubes containing EDTA. Briefly, 200 µL 1× phosphate-buffered saline (PBS) was added to the tube, followed by a short spin. DNA extraction was carried out using QIAamp DNA blood mini kit as per the manufacturer’s protocol (Qiagen, CA, USA). Then, DNA was eluted in 50 µL elution buffer and extracted DNA was stored at −20 °C for further molecular analysis.

### 4.7. Plasmodium Species Detection by Nested PCR (nPCR)

Species-specific nested PCR was performed to confirm the presence of *Plasmodium* species targeting the 18S rRNA as described by Siwal et al. (2018) [37]. For each sample, nest-1 PCR was carried out in a final volume of 20 µL reaction containing 1× Promega master mix, 0.25 µM primer, 2 mM MgCl_2_, and 5 µL of DNA template. Nest-2 PCR was carried out in a final volume of 10 µL reaction containing 1× Promega master mix, 0.5 µM primer, 2 mM MgCl_2_, and 2.8 µL of nest-1 PCR product as template. Laboratory adapted Dd2 and 3D7 *Plasmodium falciparum*, and previously confirmed positive *P. vivax* DNA samples were included as positive controls for every set of PCR. Nuclease free water and DNA of healthy individuals were also included as negative controls for the PCR assays. The amplified products were then visualized in 2% agarose gel stained with 0.5 µg/mL ethidium bromide under BioRad XR UV transilluminator.

### 4.8. Light Trap Collections

Host seeking adult female mosquitoes were collected using the CDC miniature light trap fitted in human dwellings of Dhansinghpara and Bidyapara villages. Light traps were fitted in the verandah, an outdoor porch with a roof outside the house. Collections were made monthly using five light traps in each village with two index houses fixed per month and three randomly rotated. Based on the baseline data, collections were made from two fixed houses every month and a few randomly selected houses, often those households which reported malaria cases. Traps were usually placed adjacent to the bed.

### 4.9. Mosquito Identification

*Anopheles* mosquitoes collected in the field were brought to the laboratory and identified morphologically using the keys of Nagpal and Sharma 1991 [38]. Morphologically identified specimens belonging to the Dirus and Minimus complexes were confirmed to species level by molecular methods using DNA extracted from leg or abdomen of the mosquito through allele-specific polymerase chain reaction (ASPCR) assays [39,40,41].

### 4.10. Plasmodium Species Detection by Nested Quantitative PCR (nqPCR)

The very sensitive nested real-time PCR method targeting the *Plasmodium* cytochrome *b* gene as described by Canier et al. (2013) in *Plasmodium* blood stages was performed to detect *Plasmodium* species with some modifications [42] in the known primary vectors of the area, i.e., *Anopheles minimus* and *Anopheles baimaii* [4,38]. For parasite sporozoite screening in mosquitoes, we used DNA extracted from the head and thorax dissected from a single *Anopheles* specimen for each reaction. As we obtained little DNA from the head and thorax part of a single mosquito, we used the more sensitive technique of detection by nested quantitative PCR (nqPCR) for cytochrome b gene, which has more copy numbers, and this method is more sensitive in detecting a low amount. For all the real-time assays, in a 10 µL reaction volume, Promega 2× SYBR green master mix was used with carboxy-X-rhodamine (CXR) passive dye where 2.5 µL DNA was used as a template, and for every sample, the reaction was carried out in duplicate.

### 4.11. Meteorological Data Collection

The study used rainfall data from National Aeronautics and Space Administration’s (NASA) GPM (Global Precipitation Measurement) project from January to March 2021. The dataset used is the Integrated Multi-Satellite Retrievals for GPM (IMERG) Late Daily 10 × 10 km Level-3 Integrated Multi-Satellite Retrievals for GPM Daily Late Run (GPM 3IMERGDL) of GPM Level 3, derived from the dataset of the half-hourly 3IMERGHL. The result is an accelerated late estimate of the accumulated daily precipitation. The temperature and relative humidity data were taken from NASA’s MERRA-2 (Modern-Era Retrospective analysis for Research and Applications) model. The used products are the daily maximum (T2MMAX), minimum (T2MMIN), average temperature (T2MMEAN), and relative humidity on a spatial resolution of 50 km × 62.5 km. The datasets were downloaded in NetCDF formats, which were further extracted using software codes.

### 4.12. Preparation of Ecological Maps

Land use land cover mapping was prepared using orthorectified Indian Remote Sensing satellite data, Cartosat-1 (2.5 m) and LISS-IV (5.8 m), employing on-screen visual interpretation techniques in the GIS (Geographical Information System) platform. Major land use and land cover categories and subcategories were delineated and updated using the latest data (2019) on the spatial layer initially prepared under NRSC/ISRO (National Remote Sensing Centre/Indian Space Research Organization).

Space-based Information Support for Decentralized Planning at the Panchayat level was used. The mapping was done at 1:10,000 scale. Field verifications were made by the project team to check the accuracy of the interpreted data. Geolocations of *P. vivax* positive households were collected during the survey and plotted on the map for analyses of the proximity of the households to the Jhum fields/plantations/open forests/deep forests/croplands.

### 4.13. Statistical Analysis

IBM SPSS 20 and Epi InfoTM version 7, CDC (Atlanta, GA, USA) were used for statistical analysis.

### 4.14. Malaria Case Records

Malaria yearly cases, API, and cases from January to March for Bidyapara and Dhansingh villages were compiled from the reports and registers of health workers and volunteers, which include routine active/passive surveillance and mass surveillance data. Data on annual malaria cases were taken from the reports of the Malaria Program, which include routine active/passive surveillance and mass surveillance data.

## Figures and Tables

**Table 1:** The number of cases found positive by 18S nested PCR in mass surveillance during February–March 2021 in Dhansinghpara and Bidyapara. N denotes the total no. of samples tested in each village. Species wise distribution of *P. falciparum* (*Pf*), *P. vivax* (*Pv*), and *P. falciparum* + *P. vivax* (*Pf* + *Pv*) cases are shown.

	Dhansinghpara (N = 90) (% Positive)	Bidyapara (N = 60) (% Positive)	Total Positivity (N = 150) (% Positive)
*Pf*	5 (5.5)	1 (1.6)	6 (4)
*Pv*	29 (32.2)	11 (18.3)	40 (26.7)
*Pf + Pv*	13 (14.4)	13 (21.7)	26 (17.3)
Total	47 (52.2)	25 (41.7)	72 (48.0)

**Table 2:** Age group-wise distribution of symptomatic, asymptomatic cases of *P. falciparum* (*Pf*), *P. vivax* (*Pv*), and *P. falciparum* + *P. vivax* (*Pf* + *Pv*). Species wise and total positive cases as well as negative case distribution % for each age group are given in the parentheses.

Age Groups of Tested People in Two Villages	Symptomatic Malaria Cases				Asymptomatic Malaria Cases				Total Tested	Positives (%)	Negatives (%)
	*Pf* (%)	*Pv* (%)	*Pv + Pf* (%)	Total Cases (%)	*Pf* (%)	*Pv* (%)	*Pv + Pf* (%)	Total Cases (%)			
0 to <5 years	0 (0)	1 (9.1)	0 (0)	1 (9.1)	0 (0)	4 (36.4)	1 (9.1)	5 (45.5)	11	6 (54.5)	5 (45.5)
5 to <15 years	0 (0)	1 (1.6)	3 (4.7)	4 (6.3)	3 (4.7)	14 (21.9)	9 (14)	26 (40.6)	64	30 (46.9)	34 (53.1)
<15 years and above	0 (0)	2 (2.7)	0 (0)	2 (2.7)	3 (4)	18 (24)	13 (17.3)	34 (45.3)	75	36 (48)	39 (52)
Total	0 (0)	4 (2.7)	3 (4.7)	7 (4.7)	6 (4)	36 (24)	23 (15.3)	65 (43.3)	150	72 (48)	78 (52)

**Table 3:** Age group-wise distribution of submicroscopic cases of *P. falciparum* (*Pf*), *P. vivax* (*Pv*), and *P. falciparum* + *P. vivax* (*Pf* + *Pv*).

Age Groups of Tested People in Two Villages	Sub-Microscopic Cases				
	*Pf* (%)	*Pv* (%)	*Pf + Pv* (%)	Total Cases (%)	Total Tested
0 to <5 years	0 (0)	3 (30)	1 (10.0)	4 (40)	10
5 to 15 years	1 (1.8)	14 (25.5)	4 (7.3)	19 (34.5)	55
>15 years	3 (6.1)	7 (14.3)	9 (18.4)	19 (38.8)	49
Total	4 (3.5)	23 (20.2)	15 (13.2)	42 (36.9)	114

## Data Availability

The data presented in this study are available on request from the corresponding author. The data are not publicly available due to ethical and privacy reasons.

## Supplementary Materials

The following are available online at https://www.mdpi.com/article/10.3390/pathogens10101259/s1, Table S1: Malaria yearly cases and Annual Parasite Incidence (API) along with cases of January to March for Ambassa PHC as reported by the Malaria Program, which includes routine active, passive surveillance, and mass surveillance data. Table S2: The proportion of *P. vivax* malaria cases as reported in Tripura from 2008 to 2019 (data source NVBDCP, India) (NHM Tripura Website Available online: http://tripuranrhm.gov.in/nvbdcp.html. accessed on 25 March 2015 and 3 April 2021), Figure S1: Trends of the positivity rate of *P. falciparum* *(SPF)* and *P. vivax* *(SPV).* These show that the *P. falciparum* is falling at a much faster rate than that of *P. vivax* and is now lower than *SPV*. The proportion of *P. vivax* among total malaria cases has increased. Data Source: NVBDCP India, accessed 25 June 2020 and 4 December 2020.

## References

[1] World Health Organization (2020). World Malaria Report: 20 Years of Global Progress and Challenges. World Health Organization. https://www.who.int/publications/i/item/9789240015791.

[2] National Strategic Plan for Malaria Elimination (2017-22) Launched. https://www.who.int/india/news/detail/11-07-2017-national-strategic-plan-for-malaria-elimination-.

[3] Sarmah N.P., Bhowmik I.P., Sarma D.K., Sharma C.K., Medhi G.K., Mohapatra P.K., Mahanta J., Bhattacharyya D.R. (2019). Role of Anopheles baimaii: A potential vector of epidemic outbreak in Tripura, North-east India. J. Glob. Health Rep..

[4] Babiker H.A., Abdel-Muhsin A.M., Ranford-Cartwright L.C., Satti G., Walliker D. (1998). Characteristics of *Plasmodium falciparum* parasites that survive the lengthy dry season in eastern Sudan where malaria transmission is markedly seasonal. Am. J. Trop. Med. Hyg..

[5] Ouedraogo A.L., Gonçalves B.P., Gnémé A., Wenger E.A., Guelbeogo M.W., Ouedraogo A., Gerardin J., Bever C.A., Lyons H., Pitroipa X. (2016). Dynamics of the human infectious reservoir for malaria determined by mosquito feeding assays and ultrasensitive malaria diagnosis in Burkina Faso. J. Infect. Dis..

[6] Portugal S., Tran T.M., Ongoiba A., Bathily A., Li S., Duombo S., Skinner J., Doumtabe D., Kone Y., Sangala J. (2017). Treatment of chronic asymptomatic *Plasmodium falciparum* infection does not increase the risk of clinical malaria upon reinfection. Clin. Infect. Dis..

[7] Andrade C.M., Fleckenstein H., Thomson-Luque R., Doumbo S., Lima N.F., Anderson C., Hibbert J., Hopp C.S., Tran T.M., Li S. (2020). Increased circulation time of *Plasmodium falciparum* underlies persistent asymptomatic infection in the dry season. Nat. Med..

[8] Coleman R.E., Kumpitak C., Ponlawat A., Maneechai N., Phunkitchar V., Rachapaew N., Zollner G., Sattabongkot J. (2004). Infectivity of asymptomatic Plasmodium-infected human populations to *Anopheles dirus* mosquitoes in western Thailand. J. Med. Entomol..

[9] Lindblade K.A., Steinhardt L., Samuels A., Kachur S.P., Slutsker L. (2014). The silent threat: Asymptomatic parasitemia and malaria transmission. Expert. Rev. Anti-Infect. Ther..

[10] Parker D.M., Matthews S.A., Yan G., Zhou G., Lee M.C., Sirichaisinthop J., Kirakorn K., Fan Q., Li P., Sattabongkot J. (2015). Microgeography and molecular epidemiology of malaria at the Thailand-Myanmar border in the malaria pre-elimination phase. Malar. J..

[11] Durnez L., Pareyn M., Mean V., Kim S., Khim N., Menard D., Coosemans M., Sochantha T., Sluydts V. (2018). Identification and characterization of areas of high and low risk for asymptomatic malaria infections at sub-village level in Ratanakiri, Cambodia. Malar. J..

[12] Starzengruber P., Fuehrer H.P., Ley B., Thriemer K., Swoboda P., Habler V.E., Jung M., Graninger W., Khan W.A., Haque R. (2014). High prevalence of asymptomatic malaria in south-eastern Bangladesh. Malar. J..

[13] Waite J.L., Suh E., Lynch P.A., Thomas M.B. (2019). Exploring the lower thermal limits for development of the human malaria parasite, *Plasmodium falciparum*. Biol. Lett..

[14] van Eijk A.M., Sutton P.L., Ramanathapuram L., Sullivan S.A., Kanagaraj D., Priya G.S.L., Ravishankaran S., Asokan A., Sangeetha V., Rao P.N. (2019). The burden of submicroscopic and asymptomatic malaria in India revealed from epidemiology studies at three varied transmission sites in India. Sci. Rep..

[15] Chourasia M.K., Raghavendra K., Bhatt R.M., Swain D.K., Meshram H.M., Meshram J.K., Suman S., Dubey V., Singh G., Prasad K.M. (2017). Additional burden of asymptomatic and sub-patent malaria infections during low transmission season in forested tribal villages in Chhattisgarh, India. Malar. J..

[16] Chaturvedi N., Krishna S., Bharti P.K., Gaur D., Chauhan V.S., Singh N. (2017). Prevalence of afebrile parasitaemia due to *Plasmodium falciparum* & *P. vivax* in district Balaghat (Madhya Pradesh): Implication for malaria control. Indian J. Med. Res..

[17] Ganguly S., Saha P., Guha S.K., Biswas A., Das S., Kundu P.K., Maji A.K. (2013). High prevalence of asymptomatic malaria in a tribal population in eastern India. J. Clin. Microbiol..

[18] Kumari P., Sinha S., Gahtori R., Yadav C.P., Pradhan M.M., Rahi M., Pande V., Anvikar A.R. (2020). Prevalence of asymptomatic malaria parasitemia in Odisha, India: A challenge to malaria elimination. Am. J. Trop. Med. Hyg..

[19] Dhiman S., Goswami D., Rabha B., Yadav K., Chattopadhyay P., Veer V. (2015). Absence of asymptomatic malaria in a cohort of 133 individuals in a malaria endemic area of Assam, India. BMC Public Health.

[20] Forest Survey of India. https://fsi.nic.in/isfr2019/isfr-fsi-vol2.pdf.

[21] Sharma V.P., Dev V., Phookan S. (2015). Neglected *Plasmodium vivax* malaria in northeastern States of India. Indian, J. Med. Res..

[22] Baird J.K. (2013). Evidence and implications of mortality associated with acute *Plasmodium vivax* malaria. Clin. Microbiol. Rev..

[23] Baird J.K. (2007). Neglect of *Plasmodium vivax* malaria. Trends Parasitol..

[24] Kochar D.K., Saxena V., Singh N., Kochar S.K., Kumar S.V., Das A. (2005). *Plasmodium vivax* malaria. Emerg. Infect. Dis..

[25] Mendis K., Sina B.J., Marchesini P., Carter R. (2001). The neglected burden of *Plasmodium vivax* malaria. Am. J. Trop. Med. Hyg..

[26] (2019). World Malaria Report. https://www.who.int/publications/i/item/9789241565721.

[27] Moreira C.M., Abo-Shehada M., Price R.N., Drakeley C.J. (2015). A systematic review of sub-microscopic *Plasmodium vivax* infection. Malar. J..

[28] Mueller I., Galinski M.R., Baird J.K., Carlton J.M., Kochar D.K., Alonso P.L., del Portillo H.A. (2009). Key gaps in the knowledge of *Plasmodium vivax*, a neglected human malaria parasite. Lancet Infect. Dis..

[29] Vallejo A.F., García J., Amado-Garavito A.B., Arévalo-Herrera M., Herrera S. (2016). *Plasmodium vivax* gametocyte infectivity in sub-microscopic infections. Malar. J..

[30] Krotoski W.A. (1989). The hypnozoite and malarial relapse. Prog. Clin. Parasitol..

[31] Battle K.E., Karhunen M.S., Bhatt S., Gething P.W., Howes R.E., Golding N., Van Boeckel T.P., Messina J.P., Shanks G.D., Smith D.L. (2014). Geographical variation in *Plasmodium vivax* relapse. Malar. J..

[32] Von Seidlein L., White N.J. (2021). Taking on *Plasmodium vivax* malaria: A timely and important challenge. PLoS Med..

[33] Tyrrell M.B., Krit M., Sluydts V., Tho S., Sokny M., Mean V. (2019). Kim, S.; Menard, D.; Grietens, K.P.; Abrams, S.; et al. Households or hotspots? Defining intervention targets for malaria elimination in Ratanakiri Province, Eastern Cambodia. J. Infect. Dis..

[34] Galagan S.R., Prue C.S., Khyang J., Khan W.A., Ahmed S., Ram M., Alam M.S., Haq M.Z., Akter J., Streatfield P.K. (2014). The practice of Jhumcultivation and its relationship to *Plasmodium falciparum* infection in the Chittagong hill districts of Bangladesh. Am. J. Trop. Med. Hyg..

[35] Bhowmick I.P., Nirmolia T., Sarma D.K., Gogoi K., Subbarao S., Sarma N., Senapati S., Borah L., Mohapatra P.K., Bhattacharya D.R. Unveiling the hidden low density, submicroscopic and asymptomatic malaria burden burden of malaria in Tripura in a post epidemic mass surveillance in some endemic villages of Dhalai District in 2014.

[36] World Health Organisation Malaria Parasite Counting. https://www.who.int/publications/i/item/HTM-GMP-MM-SOP-09.

[37] Siwal N., Singh U.S., Dash M., Kar S., Rani S., Rawal C., Singh R., Anvikar A.R., Pande V., Das A. (2018). Malaria diagnosis by PCR revealed differential distribution of mono and mixed species infections by *Plasmodium falciparum* and *P. vivax* in India. PLoS ONE.

[38] Nagpal B.N., Sharma V.P. (1995). Indian Anophelines.

[39] Walton C., Handley J.M., Kuvangkadilok C., Collins F.H., Harbach R.E., Baimai V., Butlin R.K. (1999). Identification of five species of the *Anopheles dirus* complex from Thailand, using allele-specific polymerase chain reaction. Med. Vet. Entomol..

[40] Phuc H.K., Bll A.J., Son L., Hanh N.V., Tu N.D., Lien N.G., Verardi A., Townson H. (2003). Multiplex PCR assay for malaria vector *Anopheles minimus* and four related species in the Myzomyia series from Southeast Asia. Med. Vet. Entomol..

[41] Singh S., Prakash A., Yadav R.N.S., Mohapatra P.K., Sarma N.P., Mahanta J., Bhattacharyya D.R. (2012). Anopheles (Cellia) maculatus group: Its spatial distribution and molecular characterization of member species in north-east India. Acta Trop..

[42] Canier L., Khim N., Kim S., Sluydts V., Heng S., Dourng D., Eam R., Chy S., Khean C., Loch K. (2013). An innovative tool for moving malaria PCR detection of parasite reservoir into the field. Malar. J..

