# Supplementary material for: Dry Post Wintertime Mass Surveillance Unearths a Huge Burden of P. vivax, and Mixed Infection with P. vivax P. falciparum, a Threat to Malaria Elimination, in Dhalai, Tripura, India
Source: Pathogens. 2021 Sep 29;10(10):1259. doi: 10.3390/pathogens10101259 (PMC8541100; doi:10.3390/pathogens10101259)
Supplement: Supplementary file 1 [file pathogens-10-01259-s001.zip › pathogens-1338115-supplementary.pdf]

Supplementary File

Table S1. Malaria yearly cases and Annual Parasite Incidence (API) along with cases of January to March for Ambassa PHC as reported by the Malaria Program, which includes routine active, passive surveillance, and mass surveillance data.

	Ambassa PHC					
	2018		2019		2020	
	Cases	API	Cases	API	Cases	API
Yearly	1670	26.2	523	8.2	322	5
Jan–Mar	38		65		9	

Table S2. Malaria yearly cases and Annual Parasite Incidence (API) along with cases of January to March and Monthly Parasite Incidence (MPI) for Bidyapara and Dhansinghpara villages. These were based on the data compiled from the reports and registers of health workers and volunteers, which include routine active, passive surveillance, and mass surveillance data.

	2018							
	Jan		Feb		March		Whole Year	
	Cases	MPI	Cases	MPI	Cases	MPI	Cases	API
Bidyapara	0	0	0	0	1	0	107	570.5
Dhansinghpara	0	0	1	4.9	2	40.9	348	26.2
	2019							
	Jan		Feb		March		Whole Year	
	Cases	MPI	Cases	MPI	Cases	MPI	Cases	API
Bidyapara	1	2.6	1	2.6	0	0	**21**	55.4
Dhansinghpara	2	3.2	4	6.5	0	0	**110**	180.3
	2020							
	Jan		Feb		March		Whole Year	
	Cases	MPI	Cases	MPI	Cases	MPI	Case	API
Bidyapara	0	0	0	0	0	0	14	36.5
Dhansinghpara	2	3.2	4	6.3	1	1.5	10	15.8

	2021					
	Jan		Feb		March	
	Cases	MPI	Cases	MPI	Cases	MPI
Bidyapara	0	0	0	0	2	2.5
Dhansinghpara	0	0	1	1.6	3	3.1

Table S2. The proportion of *P. vivax* malaria cases as reported in Tripura from 2008 to 2019 (Data source: NHM Tripura Website Available online: http://tripuranrhm.gov.in/nvbdcp.html. accessed on 25 March 2015 and 3 April 2021)

Year	*P. Vivax* Cases	*P. Vivax* Proportion (%)
2008	2306	8.9
2009	1478	6.1
2010	1131	4.7
2011	605	4.2
2012	650	5.6
2013	398	5.4
2014	1587	3.1
2015	2451	7.5
2016	1001	9.5
2017	480	6.8
2018	479	3.7
2019	801	6.4
2020	295	8.7

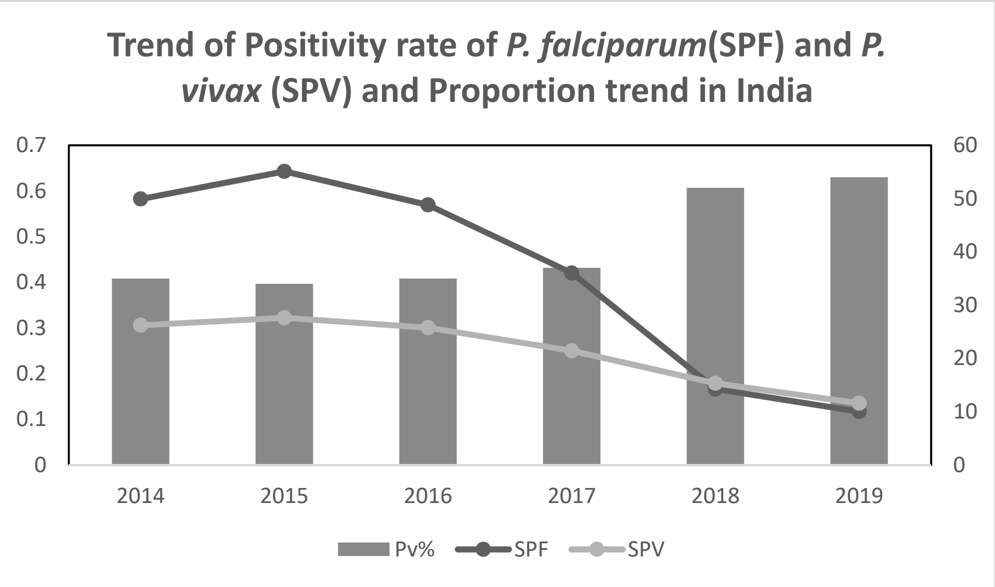

Figure S1. Trends of the positivity rate of *P. falciparum (SPF)* and *P. vivax (SPV)*. These show that the *P. falciparum* is falling at a much faster rate than that of *P. vivax* and is now lower than S*PV*. The proportion of *P. vivax* among total malaria cases has increased (Data Source: NVBDCP India, accessed 25 July 2020 and 4 December 2020).
